# A transcriptomic insight into the infective juvenile stage of the insect parasitic nematode, *Heterorhabditis indica*

**DOI:** 10.1186/s12864-016-2510-z

**Published:** 2016-03-01

**Authors:** Vishal S. Somvanshi, Shachi Gahoi, Prakash Banakar, Prasoon Kumar Thakur, Mukesh Kumar, Manisha Sajnani, Priyatama Pandey, Uma Rao

**Affiliations:** Division of Nematology, ICAR-Indian Agricultural Research Institute, New Delhi, 110012 India

## Abstract

**Background:**

Nematodes are the most numerous animals in the soil. Insect parasitic nematodes of the genus *Heterorhabditis* are capable of selectively seeking, infecting and killing their insect-hosts in the soil. The infective juvenile (IJ) stage of the *Heterorhabditis* nematodes is analogous to *Caenorhabditis elegans* dauer juvenile stage, which remains in ‘arrested development’ till it finds and infects a new insect-host in the soil. *H. indica* is the most prevalent species of *Heterorhabditis* in India. To understand the genes and molecular processes that govern the biology of the IJ stage, and to create a resource to facilitate functional genomics and genetic exploration, we sequenced the transcriptome of *H. indica* IJs.

**Results:**

The de-novo sequence assembly using Velvet-Oases pipeline resulted in 13,593 unique transcripts at N50 of 1,371 bp, of which 53 % were annotated by blastx. *H. indica* transcripts showed higher orthology with parasitic nematodes as compared to free living nematodes. *In-silico* expression analysis showed 30 % of transcripts expressing with ≥100 FPKM value. All the four canonical dauer formation pathways like cGMP-PKG, insulin, dafachronic acid and TGF-β were active in the IJ stage. Several other signaling pathways were highly represented in the transcriptome. Twenty-four orthologs of *C. elegans* RNAi pathway effector genes were discovered in *H. indica,* including *nrde-3* that is reported for the first time in any of the parasitic nematodes. An ortholog of *C. elegans tol-1* was also identified. Further, 272 kinases belonging to 137 groups, and several previously unidentified members of important gene classes were identified.

**Conclusions:**

We generated high-quality transcriptome sequence data from *H. indica* IJs for the first time. The transcripts showed high similarity with the parasitic nematodes, *M. hapla,* and *A. suum* as opposed to *C. elegans*, a species to which *H. indica* is more closely related. The high representation of transcripts from several signaling pathways in the IJs indicates that despite being a developmentally arrested stage; IJs are a hotbed of signaling and are actively interacting with their environment.

**Electronic supplementary material:**

The online version of this article (doi:10.1186/s12864-016-2510-z) contains supplementary material, which is available to authorized users.

## Background

Nematodes are the most abundant metazoans on earth and show remarkable diversity in their ecological and feeding habits [1]. Although notorious as parasites and pathogens of humans, animals, and plants, the majority of nematodes are beneficial to us as they recycle nutrients in soils and oceans [1, 2]. Another beneficial nematode group known as entomopathogenic nematodes (EPNs) encompass two genera, *Steinernema,* and *Heterorhabditis.* These EPNs symbiotically associate with gram-negative gammaproteobacteria, *Xenorhabdus,* and *Photorhabdus*, respectively [3]. Because of their ability to kill insects rapidly and amenability to mass production, they are widely used for the biological control of the insect pests of crops [4–6]. The EPNs are models to study animal-microbe symbiosis [7–10], nematode parasitism [11] and ecology [12, 13].

The infective juvenile (IJ) stage of the *Heterorhabditis* spp. is a developmentally arrested stage analogous to the dauer stage of the *C. elegans* [14], and infective L3 stage of many animal parasitic nematodes [15]. IJs are the only EPN stage found in nature outside the insect-host, and are capable of surviving tough environmental conditions in the soil for long periods of time. The nematodes in the IJ stage do not feed or grow until they find a new insect-host, and they possess a remarkable ability to actively search, follow and infect their insect-host in the soil environment [16, 17]. IJs are known to show different kinds of parasitic behaviors [18]. They can be desiccated to quiescence or frozen in liquid nitrogen [19, 20], and then be revived back to life. Thus, there is a possibility to extend the lifespan/delay life cycle. Because of this remarkable environmental toughness of the IJs, all the EPN formulations, presently available in the market, are based on this stage. An extensive body of research exists on the genes, pathways, and processes involved in aging in the free-living nematode, *C. elegans* [21–23]. A similar understanding of genes that increase the lifespan in EPNs would be directly beneficial in extending the shelf-life of EPN IJs, and IJ based formulations to improve their use as a pest control product [24–26].

Genomic tools and technologies have allowed the researchers to uncover the amazing biology of nematodes [27–29]. The genome of the EPN, *Heterorhabditis bacteriophora* TTO1-M31e strain has been sequenced [30] and is available in the public domain. Additionally, the expressed sequence tags (ESTs) of *H. bacteriophora* GPS-11 strain [31, 32] and transcriptome of the adult stage of *H. bacteriophora* TTO1-M31e were published earlier [33]. Large amount of information is available on molecular biology of the dauer/developmentally arrested L2 and L3 stages of various nematodes, such as free-living *C. elegans* and *C. briggsae* [34–38], insect-associated *Pristionchus* [39, 40], animal parasitic *Strongyloides stercoralis* [41] and *Ostertagia ostertagi* [42] and many plant parasitic nematodes [43–48]. However, such information is completely lacking for IJ stage of EPNs. Scanty information available on the *Heterorhabditis* IJ ‘recovery’ is not adequate to decipher the various molecular and physiological pathways specific to these IJs [33, 49]. Additionally, it is suggested that genes expressed in survival or dispersal stages in nematodes, such as dauer, and EPN IJs, are more likely to be novel, compared with the genes expressed in adult or larval stages [29].

*H. indica* was the first species of this genus recorded from India [50]. Since then, various surveys showed that *H. indica* is the most predominant species of Heterorhabditid nematode in India and is found in almost all the geographical parts of the country. Therefore, *H. indica* is naturally suitable for incorporation in insect biological control programs in India. In the present study, the transcriptomic analysis of the IJ stage of *H. indica* was carried out to understand the molecular processes and pathways active at this stage, and to create a resource for further functional genomics and genetic investigations.

## Results

### Transcriptome sequencing and assembly

The mRNA sequencing of IJ stage of *H. indica* using the Illumina GAIIx platform yielded about 51.2 million reads of 100 base read-lengths generating 64x coverage. After quality filtering, 42.3 million high-quality reads totalling 4.2 gigabases of data were obtained. The de-novo sequence assembly was carried out by Velvet at different k-mer lengths (51–93 with step size of 4) with minimum contig length of 200. The optimal assembly was attained at k-mer 83 which resulted in 18,710 contigs with 909 bp N50 (Table 1). Merging of transcripts from 71 to 83 k-mer range by Oases resulted in 23,827 transcripts with 1,292 bp N50 size. Removing duplicates by cd-hit-est, and filtering out < 300 bp transcripts resulted in 13,593 unique transcripts with N50 of 1,371 bp (Table 1). Total of 13,592 proteins were predicted by ORFPredictor [51] which were then used for downstream analysis.

**Table 1:** Assembly statistics of *H. indica* transcriptome generated by Velvet and Oases

Assembly statistics of *H. indica* transcriptome generated by velvet				
k-mer length	71	75	79	83
No. of contig	22,698	21,760	20,363	18,710
Min contig length (bp)	200	200	200	200
Max contig length (bp)	12,876	11,783	10,340	10,673
N50 (bp)	810	828	872	909
Assembly statistics of *H. indica* transcriptome generated by Oases				
Parameters	Reading			
No. of transcripts	23,827			
Total assembly (Mb)	22			
Min transcript length (bp)	102			
Max transcript length (bp)	12,876			
N50	1,292			
Final assembly statistics of the *H. indica* transcriptome after Velvet-Oases pipeline, cd-hit-est and filtering for <300 bp reads				
Parameters	Reading			
No. of unique transcripts	13,593			
Total assembly (Mb)	15			
min transcript length (bp)	300			
Max transcript length (bp)	12,876			
N50	1,371			

### Characterization of *H. indica* transcripts

**Fig. 1:**
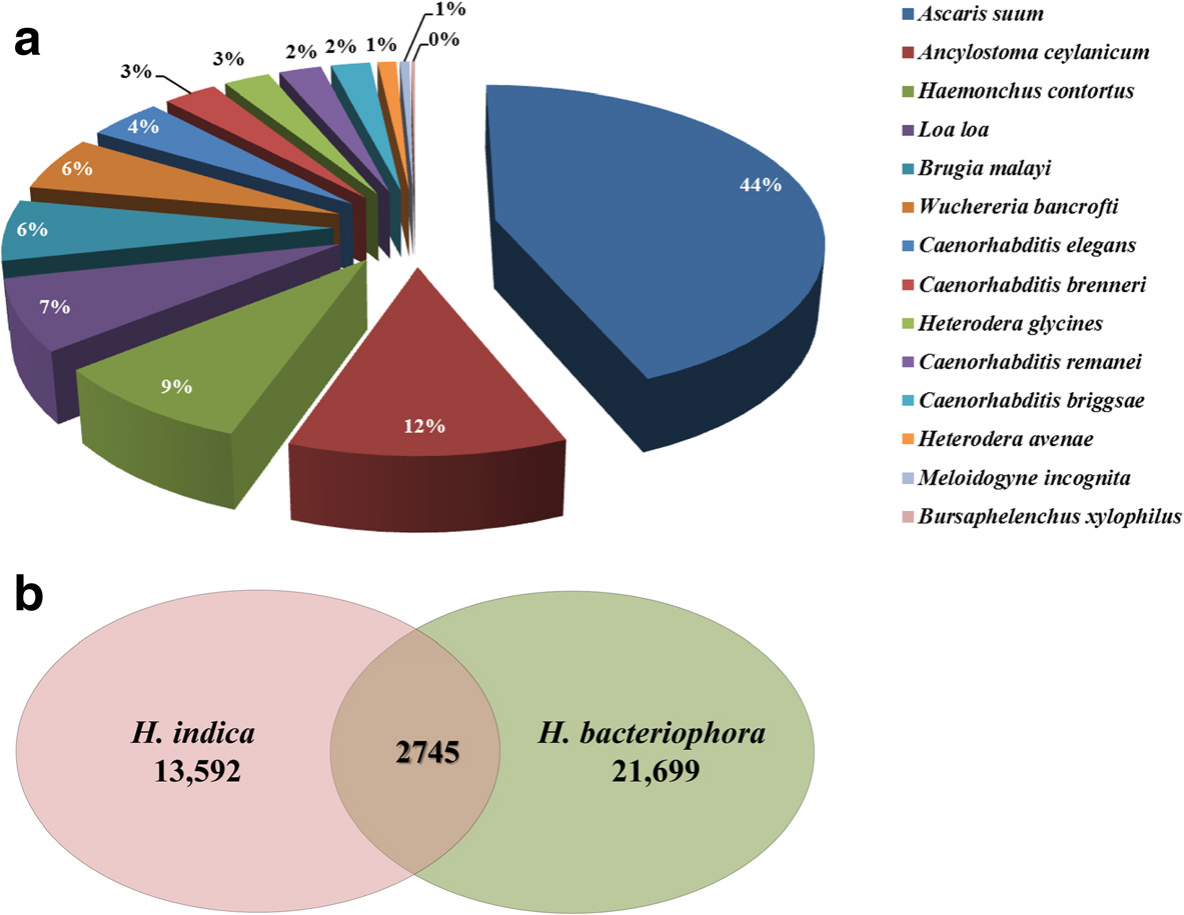
**a** Distribution of the top 10 nematode species with most homologs to *Heterorhabditis indica.* The distribution was calculated using best blastx hits. **b**. Venn diagram of *H. indica* transcripts matching *H. bacteriophora* proteins in a standalone blast

The blastx analysis of *H. indica* transcripts resulted in annotation of 7,246 transcripts (Additional file 1: Table S1a), of which 6,320 hits matched to animal and plant parasitic, as well as free-living nematodes i.e. *A. suum* (2,763 hits), *Ancylostoma ceylanicum* (741 hits), *Haemonchus contortus* (558 hits), *Loa loa* (466 hits), *Brugia malayi* (397 hits), *Wucheria bancroftii* (357 hits), *C. elegans* (269 hits), *C. brenneri* (193 hits), *Heterodera glycines* (167 hits), *C. remanei* (153 hits), *C. briggsae* (141 hits), *H. avenae* (67 hits), *M. incognita* (35 hits), *Bursaphelenchus xylophilus* (13 hits) (Fig. 1a). Due to absence of *H. bacteriophora* hits in the blastx results, we performed a standalone blastx of *H. indica* transcripts against *H. bacteriophora* protein dataset (PRJNA13977) downloaded from the wormbase (http://parasite.wormbase.org/ftp.html). The blastx resulted in 2,745 protein hits (Fig. 1b, Additional file 2: Table S1b).

**Fig. 2:**
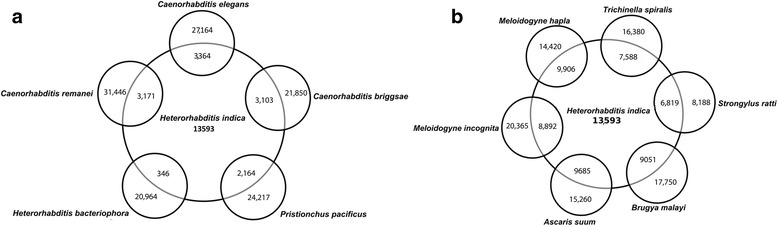
**a** *H. indica* orthologs present in selected completely sequenced genomes of free-living nematodes *C. elegans, C. remanei, C. briggsae, Pristionchus pacificus* and *Heterorhabditis bacteriophora.* **b** *H. indica* orthologs in animal and plant parasitic nematodes

Comparison of the transcripts with complete genomes of other closely related rhabditid nematodes through reciprocal blast approach showed 3,364 orthologs of *C. elegans*, 3,103 of *C. briggsae*, 3,171 of *C. remanei*, 2,164 of *P. pacificus* and 346 of *H. bacteriophora* (Fig. 2a). However, higher numbers of orthologs were identified when the transcripts were compared to the animal parasitic nematodes-9,685 orthologs in *A. suum,* 6,819 in *Strongyloides ratti* while other parasites like *Meloidogyne hapla*, *M. incognita*, *B. malayi* and *Trichinella spiralis* ranked in between these two nematodes (Fig. 2b).

### Putative functional classification using gene ontology and KEGG pathway analysis

**Fig. 3:**
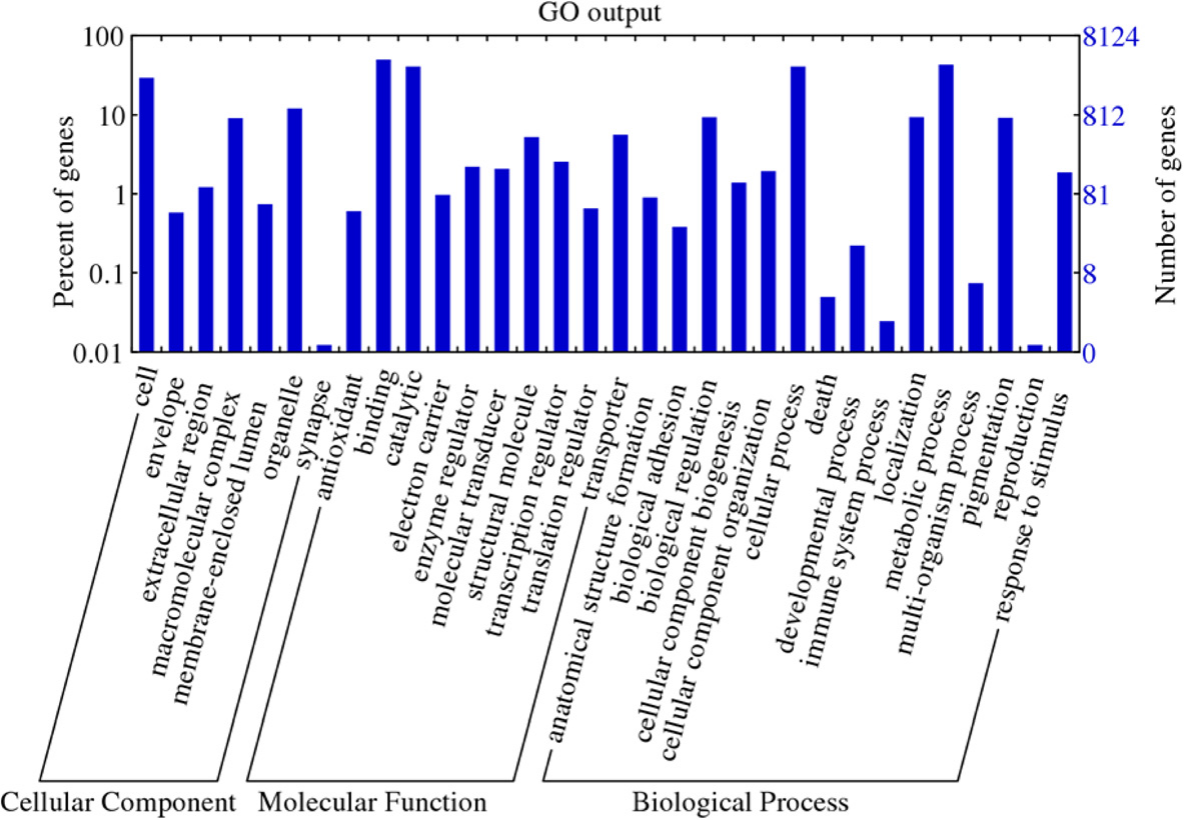
GO term analysis for all predicted proteins in IJ transcriptome of *H. indica*

All the transcripts were further functionally characterized into GO categories such as molecular functions, biological processes and cellular components. GO terms were assigned to 8,124 transcripts (Table 2, Fig. 3) of which 49.6 % (4,027) belonged to the binding category (GO:0005488) and 40.5 % (3,293) belonged to the catalytic activity of the molecular functions group (GO:0003824). Protein binding and nucleotide binding subcategories contributed 16.5 and 15.1 %, respectively, in the binding category, whereas hydrolase (14.1 %) and transferase (11.8 %) were the two most dominant subcategories in catalytic activity. The transcription regulator activity (GO:0030528) and translation regulator activity (GO:0045182) contributed 2.5 % and 0.7 % transcripts, respectively. In the biological process, 42.7 % (3,466) transcripts were grouped under metabolic processes (GO:0008152), and 40.5 % (3,293) under cellular processes (GO:0009987) (Table 2, Fig. 3). Other categories were biological regulation (GO:0065007; 9.4 %) transcripts, and stimulus (GO:0050896; 1.9 %) transcripts. Interestingly, developmental process (GO:0032502) showed only 0.2 % of the genes, while two transcripts for immune system process (GO:0002376), and one transcript each for reproduction (GO:0000003) and reproductive processes (GO:0022414) were obtained. Within the cellular component category, cell (GO:0005623;29.1 %), and organelle (GO:0043226;12.1 %) showed the maximum number of hits (Table 2).

**Table 2:** Gene ontology analysis of proteins, conceptually translated from contigs of *H. indica*

GO category	GO code	GO term	No. of proteins (%)
Cellular component	GO:0005623	Cell	2368 (29.1)
	GO:0043226	Organelle	981 (12.1)
	GO:0032991	Macromolecular complex	741 (9.1)
	GO:0005576	Extracellular region	99 (1.2)
	GO:0031974	Membrane-enclosed lumen	60 (0.7)
	GO:0031975	Envelope	47 (0.6)
	GO:0045202	Synapse	1 (0)
Molecular function	GO:0005488	Binding	4027 (49.6)
	GO:0003824	Catalytic activity	3293 (40.5)
	GO:0005215	Transporter activity	456 (5.6)
	GO:0005198	Structural molecule activity	427 (5.3)
	GO:0030528	Transcription regulator activity	207 (2.5)
	GO:0030234	Enzyme regulator activity	179 (2.2)
	GO:0060089	Molecular transducer activity	169 (2.1)
	GO:0009055	Electron carrier activity	79 (1)
	GO:0045182	Translation regulator activity	53 (0.7)
	GO:0016209	Antioxidant activity	49 (0.6)
Biological process	GO:0008152	Metabolic process	3466 (42.7)
	GO:0009987	Cellular process	3293 (40.5)
	GO:0051179	Localization	763 (9.4)
	GO:0065007	Biological regulation	762 (9.4)
	GO:0043473	Pigmentation	748 (9.2)
	GO:0016043	Cellular component organization	158 (1.9)
	GO:0050896	Response to stimulus	152 (1.9)
	GO:0044085	Cellular component biogenesis	113 (1.4)
	GO:0010926	Anatomical structure formation	73 (0.9)
	GO:0032501	Multicellular organismal process	31 (0.4)
	GO:0022610	Biological adhesion	31 (0.4)
	GO:0032502	Developmental process	18 (0.2)
	GO:0051704	Multi-organism process	6 (0.1)
	GO:0016265	Death	4 (0)
	GO:0002376	Immune system process	2 (0)
	GO:0000003	Reproduction	1 (0)
	GO:0022414	Reproductive process	1 (0)
	GO:0016032	Viral reproduction	1 (0)

**Fig. 4:**
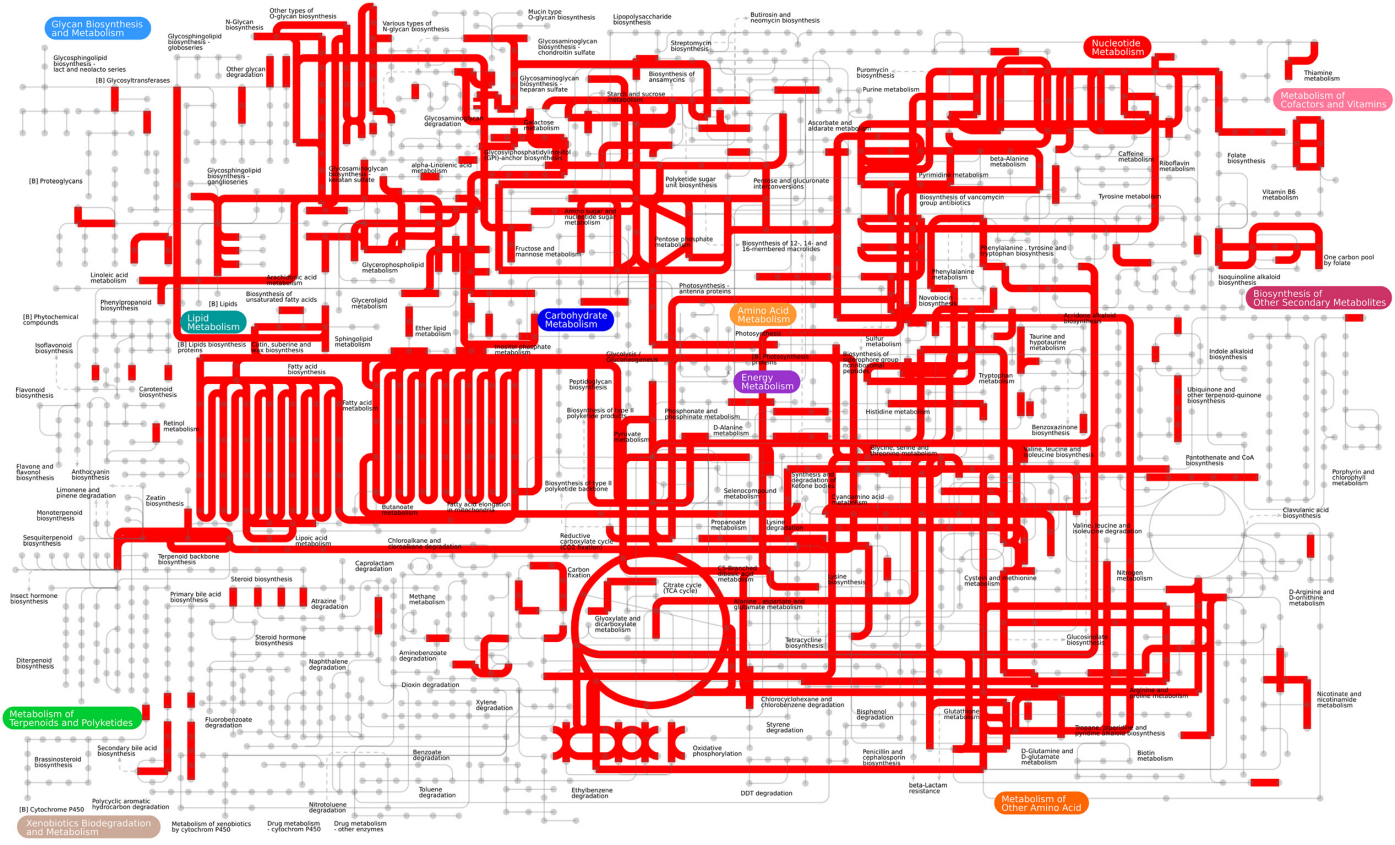
Metabolic pathways active in *H. indica* infective juveniles as revealed by the transcriptomic analysis using iPATH2 interactive pathway explorer

The transcripts were analysed to identify the key metabolic pathways and processes of which 4,738 proteins were mapped to various pathways (Table 3). The 60 most represented pathways included signaling pathways like PI3K-Akt, MAPK, Rap1, Ras, insulin, FoxO, AMPK, cAMP, Wnt, Hippo, chemokine, neurotrophin, sphingolipid, oxytocin, thyroid hormone, cGMP-PKG, and signaling pathways regulating pluripotency of stem cells (Table 3). Transcripts that were mapped to all the pathways in *H. indica* IJs are represented in Fig. 4.

**Table 3:** The sixty pathways most represented in the transcriptome of *H. indica* IJs

Pathway ID	Pathway term	No of proteins
3010	Ribosome	117
3040	Spliceosome	95
4141	Protein processing in endoplasmic reticulum	88
3013	RNA transport	87
5200	Pathways in cancer	86
5016	Huntington's disease	85
190	Oxidative phosphorylation	74
5010	Alzheimer's disease	73
230	Purine metabolism	70
4144	Endocytosis	70
5012	Parkinson's disease	68
5169	Epstein-Barr virus infection	68
5166	HTLV-I infection	67
1200	Carbon metabolism	63
4932	Non-alcoholic fatty liver disease, NAFLD	63
4120	Ubiquitin mediated proteolysis	62
5203	Viral carcinogenesis	61
4110	Cell cycle	59
240	Pyrimidine metabolism	58
5205	Proteoglycans in cancer	55
3008	Ribosome biogenesis in eukaryotes	53
4151	PI3K-Akt signaling pathway	53
3015	mRNA surveillance pathway	52
4010	MAPK signaling pathway	52
4111	Cell cycle-yeast	48
4015	Rap1, signaling pathway	47
4510	Focal adhesion	47
4014	Ras signaling pathway	46
4810	Regulation of actin cytoskeleton	46
4910 (ko04910)	Insulin signaling pathway	46
4142	Lysosome	45
4068	FoxO signaling pathway	42
4152	AMPK signaling pathway	42
4114	Oocyte meiosis	42
3018	RNA degradation	41
4024	cAMP signaling pathway	41
4310	Wnt signaling pathway	39
4390	Hippo signaling pathway	39
5206	MicroRNAs in cancer	38
1230	Biosynthesis of amino acids	37
4530	Tight junction	37
4062	Chemokine signaling pathway	37
4722	Neurotrophin signaling pathway	37
4146	Peroxisome	36
4113	Meiosis-yeast	36
5168	Herpes simplex infection	36
564	Glycerophospholipid metabolism	35
4071	Sphingolipid signaling pathway	35
4145	Phagosome	35
4914	Progesterone-mediated oocyte maturation	35
4921	Oxytocin signaling pathway	35
4919	Thyroid hormone signaling pathway	35
3420	Nucleotide excision repair	32
4022 (ko04022)	cGMP-PKG signaling pathway	32
4550	Signaling pathways regulating pluripotency of stem cells	32
3050	Proteasome	31
4721	Synaptic vesicle cycle	30
510	N-Glycan biosynthesis	29
3022	Basal transcription factors	29
5100	Bacterial invasion of epithelial cells	29

The transcripts were also analyzed using the EuKaryotic Orthologous Groups (KOG) and Protein K(c)lusters (PRK) databases. The results of the analysis are presented in Additional file 1: Table S1. The KOG analysis is a eukaryote-specific version of the Clusters of Orthologous Groups (COG) tool for identifying ortholog and paralog proteins. Broadly, 1,519 transcripts were classified to signal transduction (KOG function ID-T), 985 to transcription (KOG function ID-K), 747 to translation, ribosomal structure and biogenesis (KOG function ID-J), 566 to RNA processing and modification (KOG function ID-A), 85 to defence mechanisms (KOG function ID-V) amongst other KOG classes (Additional file 1: Table S1). A total of 3,594 transcripts were annotated using PRK database (Additional file 1: Table S1).

### Transcriptome quantitation and enrichment of significant biological categories and KEGG pathways

To get an estimate of transcript abundance, *in silico* quantitation of transcripts was done by mapping the reads from individual libraries to the non-redundant set of 13,593 transcripts using TopHat, and transcript abundance were calculated using Cufflinks. The FPKM (Fragments Per Kilobase of transcript per Million mapped reads) values for all the transcripts are given in Additional file 3: Table S2. The highly abundant transcripts were searched against KOG and PRK databases to identify their functions. We identified 202 transcripts showing ≥1000 FPKM, and 4,124 transcripts with ≥100 FPKM (Additional file 3: Table S2). The KOG analysis predicted functions for 76 proteins with ≥ 1,000 FPKM values, of which three most abundant protein classes were translation, ribosomal structure and biogenesis (KOG function ID-J), post translational modification, protein turnover, chaperones (KOG function ID-O) and intracellular trafficking, secretion, and vesicular transport (KOG function ID-U) (Table 4, Additional file 3: Table S2). In the 2,345 proteins with ≥ 100 FPKM values (Additional file 3: Table S2), other predominant protein functional classes that showed up in 2,345 proteins with ≥ 100 FPKM values were signal transduction (KOG function ID-T), energy production and conversion (KOG function ID-C), RNA processing and modification (KOG function ID-A), and transcription (KOG function ID-K). The PRK database analysis showed a similar result (Additional file 3: Table S2).

**Table 4:** KOG analysis of genes with FPKM Values ≥ 1000 and ≥100

KOG function	Function ID	Gene count
*FPKM >1000*		
Translation, ribosomal structure and biogenesis	J	27
Posttranslational modification, protein turnover, chaperones	O	10
Multiple classes		9
Intracellular trafficking, secretion, and vesicular transport	U	7
Lipid transport and metabolism	I	7
Cytoskeleton	Z	4
Defense mechanisms	V	2
Energy production and conversion	C	2
General function prediction only	R	2
Transcription	K	2
Carbohydrate transport and metabolism	G	1
Chromatin structure and dynamics	B	1
Extracellular structures	W	1
Inorganic ion transport and metabolism	P	1
Grand total		76
*FPKM >100*		
Posttranslational modification, protein turnover, chaperones	O	279
Multiple classes		264
General function prediction only	R	235
Translation, ribosomal structure and biogenesis	J	189
Function unknown	S	160
Intracellular trafficking, secretion, and vesicular transport	U	155
Signal transduction mechanisms	T	131
Energy production and conversion	C	124
RNA processing and modification	A	119
Transcription	K	108
Cytoskeleton	Z	90
Lipid transport and metabolism	I	84
Carbohydrate transport and metabolism	G	77
Amino acid transport and metabolism	E	66
Inorganic ion transport and metabolism	P	53
Cell cycle control, cell division, chromosome partitioning	D	39
Replication, recombination and repair	L	36
Nucleotide transport and metabolism	F	25
Chromatin structure and dynamics	B	24
Secondary metabolites biosynthesis, transport and catabolism	Q	21
Extracellular structures	W	18
Coenzyme transport and metabolism	H	18
Cell wall/membrane/envelope biogenesis	M	15
Defense mechanisms	V	13
Nuclear structure	Y	1
Cell motility	N	1
Grand total		2345

Metabolic pathway analysis was done using KEGG Automatic Annotation Server against *C. elegans*, *C. briggsae*, *B. malayi*, *Loa loa* and *Trichinella spiralis* pathways. The analysis of KEGG pathways represented by the abundant transcripts revealed that, among others, at FPKM ≥ 1,000, the various signaling pathways like PI3K-Akt, Hippo, HIF-signaling pathway, Rap, MAPK, calcium, sphingolipid, cGMP-PKG, insulin signaling pathway were represented by at least one or more protein (Additional file 4: Table S3). However, at FPKM ≥ 100, in addition to the above pathways, several other signaling pathways like FoxO, cAMP, Ras, sphingolipid, epithelial cell, AMPK, TGF-ß were detected (Additional file 4: Table S3).

### The kinome of *H. indica* IJs

The kinome analysis was done to identify the protein kinases important in signal transduction in all the above mentioned signaling pathways that regulate metabolism, cell cycle, growth and development, and responses to environmental stimuli. As against 438 kinases reported from *C. elegans* [52], we detected 272 in *H. indica* IJ transcriptome at stringent blastp parameters of at least 40 % sequence identity and 50 % query coverage (Table 5). These 438 (*C. elegans*) kinases were classified into 187 groups, and we found that 137 kinase groups were common between *C. elegans* and *H. indica*, whereas, 50 kinase groups were not found in *H. indica*. The details of kinase groups common between *C. elegans,* and *H. indica* are given in Table 5, and kinases that could not be discovered in *H. indica* but present in *C. elegans* are listed in Additional file 5: Table S4.

**Table 5:** Kinases belonging to different group/family/subfamily represented in IJ stage of *H. indica*

S. No.	Kinase group/family/subfamily	*C. elegans*	*H. indica*	S. No.	Kinase group/family/subfamily	*C. elegans*	*H. indica*
1	AGC/Akt	2	1	71	CMGC/DYRK/DYRK1	1	1
2	AGC/DMPK/GEK	1	3	72	CMGC/DYRK/DYRK2	3	1
3	AGC/DMPK/ROCK	1	2	73	CMGC/DYRK/PRP4	1	1
4	AGC/GRK/BARK	1	3	74	CMGC/GSK	7	4
5	AGC/GRK/GRK	1	2	75	CMGC/MAPK/ERK1	1	1
6	AGC/MAST/MAST	1	3	76	CMGC/MAPK/ERK7	1	1
7	AGC/NDR/LATS	1	1	77	CMGC/MAPK/JNK	5	1
8	AGC/NDR/NDR	1	2	78	CMGC/MAPK/nmo	1	1
9	AGC/PDK1	2	1	79	CMGC/MAPK/MAPK-Unclassified	3	2
10	AGC/PKA	2	4	80	CMGC/MAPK/p38	3	2
11	AGC/PKC/PKCa	1	3	81	CMGC/SRPK	1	1
12	AGC/PKC/PKCd	1	1	82	Other/Aur	2	2
13	AGC/PKC/PKCh	1	1	83	Other/BUB	1	1
14	AGC/PKC/PKCi	1	1	84	Other/Bud32	1	2
15	AGC/PKG	2	3	85	Other/Haspin	13	1
16	AGC/PKN	1	1	86	Other/NAK/BIKE	1	2
17	AGC/RSK/MSK	1	1	87	Other/NEK/NEK6	1	2
18	AGC/RSK/RSKp70	2	1	88	Other/NEK/NEK8	2	2
19	AGC/RSK/RSKp90	1	1	89	Other/NKF2	1	1
20	AGC/SGK	1	1	90	Other/NRBP	1	3
21	AGC/YANK	1	1	91	Other/Other-Unique	10	2
22	Atypical/ABC1/ABC1-A	1	1	92	Other/PEK/GCN2	1	2
23	Atypical/ABC1/ABC1-B	1	1	93	Other/PLK/PLK1	3	1
24	Atypical/BRD	3	3	94	Other/SCY1	2	4
25	Atypical/PDHK/PDHK	2	2	95	Other/TLK	1	3
26	Atypical/PIKK/FRAP	1	3	96	Other/ULK/ULK	2	1
27	Atypical/PIKK/SMG1	1	2	97	Other/WEE/Myt1	2	1
28	Atypical/PIKK/TRRAP	1	1	98	Other/WNK	1	2
29	Atypical/RIO/RIO1	1	2	99	Other/Worm3	2	1
30	Atypical/RIO/RIO2	1	1	100	RGC/RGC	27	13
31	Atypical/TAF1	1	3	101	STE/STE11/ASK	1	3
32	CAMK/CAMK1	1	2	102	STE/STE11/MEKK4	1	1
33	CAMK/CAMK2	1	1	103	STE/STE20/FRAY	1	2
34	CAMK/CAMKL/AMPK	2	1	104	STE/STE20/KHS	1	1
35	CAMK/CAMKL/LKB	1	2	105	STE/STE20/MSN	1	3
36	CAMK/CAMKL/MARK	2	2	106	STE/STE20/MST	1	2
37	CAMK/CAMKL/MELK	1	2	107	STE/STE20/PAKA	2	3
38	CAMK/CAMKL/NIM1	1	2	108	STE/STE20/SLK	1	3
39	CAMK/CAMKL/QIK	1	2	109	STE/STE20/TAO	1	1
40	CAMK/CAMKL/SNRK	1	1	110	STE/STE20/YSK	1	1
41	CAMK/CASK	1	1	111	STE/STE7/MEK1	1	1
42	CAMK/DAPK/DAPK	1	2	112	STE/STE7/MEK3	1	1
43	CAMK/DCAMKL	2	1	113	STE/STE7/MEK4	3	1
44	CAMK/MAPKAPK/MK2	2	2	114	STE/STE7/MEK7	2	1
45	CAMK/MAPKAPK/MNK	1	1	115	TK/Abl	1	2
46	CAMK/MLCK	4	17	116	TK/Ack	2	4
47	CAMK/PHK	1	2	117	TK/ALK	1	1
48	CAMK/PIM	2	1	118	TK/Csk	1	1
49	CAMK/PKD	2	4	119	TK/DDR	2	2
50	CAMK/PSK	1	1	120	TK/Eph	1	1
51	CAMK/TSSK	3	2	121	TK/Fer	38	4
52	CK1/CK1/CK1-A	1	1	122	TK/FGFR	1	1
53	CK1/CK1/CK1-D	1	1	123	TK/InsR	1	3
54	CK1/CK1/CK1-G	1	1	124	TK/KIN6	5	2
55	CK1/CK1-Unique	6	1	125	TKL/IRAK	1	1
56	CK1/TTBK	1	1	126	TKL/LRRK	1	2
57	CK1/TTBKL	31	4	127	TKL/MLK/HH498	1	1
58	CK1/Worm6	28	15	128	TKL/MLK/ILK	1	1
59	CMGC/CDK/CDC2	2	3	129	TKL/MLK/MLK	1	1
60	CMGC/CDK/CDK4	1	1	130	TKL/RAF/RAF	2	1
61	CMGC/CDK/CDK5	1	2	131	TKL/STKR/STKR1	2	2
62	CMGC/CDK/CDK7	1	1	132	TK/Src/Src-Unclassified	2	2
63	CMGC/CDK/CDK8	1	1	133	TKL/STKR/STKR2	1	2
64	CMGC/CDK/CDK9	1	1	134	TK/Met	2	2
65	CMGC/CDK/CRK7	1	1	135	TK/Ror	1	1
66	CMGC/CDK/PCTAIRE	1	2	136	TK/Src/Frk	1	1
67	CMGC/CDK/PFTAIRE	1	2	137	TK/TK-Unique	6	2
68	CMGC/CDK/PITSLRE	2	1				
69	CMGC/CK2	1	1				
70	CMGC/CLK	3	1				

### The secretome of *H. indica* IJs

A total of 2,374 secreted proteins were predicted (Additional file 6: Table S5a). The important proteins found in the analysis were related to neuropeptide signaling, for example, 2 each of GPCR-Family 2 like and GPCR rhodopsin-like including GPCR rhodopsin-like 7TM, and GPCR Family 3 C-terminal domains. Several hydrolases were identified, including 33 hydrolases belonging to small GTPases, glycoside hydrolases, transthyretin/hydroxyisourate hydrolase, alpha/beta hydrolase and epoxide hydrolase. The secretome showed the presence of a large contingent of peptidases that have a known role in degrading insect tissues. We could identify 38 peptidases belonging to different classes, such as metallopeptidases, trypsin-like cysteine/serine peptidases, cysteine peptidases, peptidase S1 (serine endopeptidases), S1A, S8, S10, S24, S26, S28, S53, S54, M10, M13, M14, M28, M12, M41. Some of these peptidases like carboxypeptidase possess regulatory domains. A search of the MEROPS database [53] for identification of putative peptidases (proteases, proteinases, and proteolytic enzymes) identified 64 known peptidases of the different parasitic and free-living nematodes (Additional file 7: Table S5b). Five transcription factors including STAT, p53, TFIID were also identified. Several genes involved in signaling, such as 13 members of protein kinases were present in the secreted contingent, including serine threonine, tyrosine, and thiamine phosphate kinase. Similarly, 12 members of phosphatases were found. Lastly, the transcripts showed the presence of several known stress response genes such as glutathione peroxidases, heat shock protein 70 and heat shock protein 90.

### Repeat elements in *H. indica* transcriptome

The transcriptome data was used to analyze the repeat elements because no information is available for repeat elements in this species. Transcript sequences were examined for the presence of repeat elements using Repeat Masker v-4.0.5 program. Approximately 1.4 % of the total transcripts were found to be encoded by different repetitive elements, of which 1.21 % belonged to simple repeats, and 0.29 % were low complexity repeats (Additional file 8: Table S6a). A total of 31 retroelements were found in the transcripts, with four long interspersed repeat elements (LINEs), although no short interspersed repeat elements (SINEs) were found. Among retroelements, 27 long terminal repeats (LTR) were found which was higher than non-LTR elements. Also, 15 DNA transposons of different classes, 103 small RNA, and three satellites were found (Additional file 8: Table S6a).

Using MISA to identify short sequence repeats (SSRs) revealed 2,968 sequences showing the presence of 3,635 SSRs. Out of the 2,968 sequences, 465 sequences contained more than one SSRs and 209 SSRs were present in compound formation (Additional file 9: Table S6b). Mononucleotide repeats (46.6 %), and trinucleotide repeats (46.05 %) represented the largest fraction of SSRs, followed by di-nucleotide repeats (6.3 %). The number of tetra-(32), penta-(5) and hexa-(1) nucleotide repeats were below 0.1 % (Additional file 9: Table S6b).

### RNAi pathway genes and other gene classes in *H. indica* IJs

*C. elegans* genome encodes 77 RNAi pathway effector genes, which is the most number of RNAi pathway effector genes discovered in any nematode [54]. We could identify 24 RNAi pathway effector genes in the present transcriptome (Table 7). Different RNAi effector genes identified were six genes encoding for small RNA biosynthetic proteins, four genes for dsRNA uptake, spreading and siRNA amplification, three for Argonautes, two each for RNA-induced silencing complex genes (RISC) and RNAi inhibitors, and seven for nuclear RNAi effectors (Table 6, Additional file 10: Table S7). The presence of *nrde-3* in *H. indica* (percent identity, 30.27; query coverage, 98; E-value, 1.00E-21)*,* which is responsible for nuclear translocation of RNAi triggers in *C. elegans*, is recorded for the first time in any parasitic nematode.

**Table 6:** RNAi effector genes discovered in the IJ stage of the *H. indica*

S. No.	*C. elegans* ortholog	*H. indica* IJ	*H. bacteriophora*
*Small RNA biosynthetic proteins*			
Total	10	6	2
1	*drh-3*	+	-
* 2*	*drsh-1*	+	+
* 3*	*xpo-1*	+	-
* 4*	*xpo-2*	+	-
* 5*	*dcr-1*	+	+
* 6*	*drh-1*	+	-
*dsRNA uptake and spreading and siRNA amplification effectors*			
Total	12	4	5
7	*smg-2*	+	+
8	*smg-6*	+	-
9	*ego-1*	+	+
10	*smg-5*	-	+
11	*rsd-3*	+	+
12	*sid-1*	-	+
*Argonautes (AGOs)*			
Total	*28*	3	1
13	*alg-1*	+	-
14	*rde-1*	-	+
15	*ppw-2*	+	-
16	*nrde-3*	+	-
*RNA-induced Silencing Complex (RISC) components*			
Total	4	2	2
17	*tsn-1*	+	+
18	*ain-1a*	+	-
19	*vig-1*	-	+
*RNAi inhibitors*			
Total	9	2	0
20	*eri-1*	+	-
21	*xrn-2*	+	-
*Nuclear RNAi effectors*			
Total	15	7	2
22	*mut-7*	+	-
23	*cid-1*	+	-
24	*gfl-1*	+	+
25	*mes-2*	+	-
26	*rha-1*	+	-
27	*zfp-1*	+	+
28	*mut-2*	+	-

Additionally, the *H. indica* transcriptome was analysed for presence of members of functionally important gene classes like neuropeptides (FMRFamide-related peptides (*flp)*, non-insulin, non-FMRFamide-related neuropeptide-like proteins (*nlp*), uncoordinate (*unc*), dauer formation (*daf*), fatty acid and retinol binding protein (*far*), nuclear hormone receptor (*nhr*), C-type lectin domain containing proteins (*lec*), lysozymes (*lys*) and lethal (*let*) gene classes at two stringency levels of 25 and 30 % sequence similarity and query coverage. The results are presented in Table 7. Interestingly, we also found an ortholog of *C. elegans tol-1* in the transcriptome of *H. indica* IJs (32.9 % identity, 88 query coverage at 2e–180).

**Table 7:** Members of *flp*, *nlp*, *unc*, *ins*, *daf*, *far, nhr, lec, let* and *lys* gene class present in the transcriptome of *H. indica* IJs. Gene counts for each gene class for *C. elegans* were taken from wormbase

Gene class	*C. elegans* (gene count)	*H. indica* (gene count)	
		At ≥ 25 % sequence similarity and query coverage	At ≥ 30 % sequence similarity and query coverage
*flp*	31	25	22
*nlp*	44	35	25
*unc*	111	77	69
*ins*	39	33	18
*daf*	34	24	21
*far*	8	4	0
*nhr*	283	98	36
*lec*	12	7	6
*let*	642	15	13
*lys*	10	0	0

## Discussion

The transcriptome sequencing and assembly of *H. indica* IJs resulted in 13,593 unique, high-quality transcripts at N50 value of 1,371 bp. Further, 6,320 out of 13,593 (53 %) transcripts could be annotated by blastx against nr database. Most of the blastx hits showed similarity with *A. suum* and not *H. bacteriophora* which is a closely related species. This anomaly may be attributed to the absence of *H. bacteriophora* sequences from nr database. Standalone blast identified 2,745 hits with *H. bacteriophora*.

The free living-developmentally arrested infective stage is characteristic of many parasitic nematodes [55–58]. The “dauer hypothesis” proposes that similar molecular mechanisms regulate the developmental arrest and activation of both *C. elegans* dauer larvae and analogous developmentally arrested 3^rd^ stage larvae (L3i) of parasitic nematodes [56, 57, 59] despite their evolutionary divergence [60, 61]. In the free-living model nematode, *C. elegans*, a developmentally arrested dauer stage is formed during conditions of low food abundance, high temperature [62], high dauer pheromone levels [63, 64] and high population density [65, 66]. The *daf* (abnormal dauer formation) genes identified in *C. elegans* that are involved in formation and regulation of dauer stages are placed into four dauer pathways-a cyclic guanosine monophosphate (cGMP) signaling pathway, an insulin/IGF-1-like signaling (IIS) pathway regulated by insulin-like peptide (ILP) ligands, a dauer transforming growth factor-β (TGF-β) pathway regulated by the Ce-DAF-7 ligand, and a nuclear hormone receptor (NHR) regulated by a class of steroid ligands known as dafachronic acids (DAs) [35]. Epistatic analysis revealed that the cGMP signaling pathway operates upstream of the parallel IIS and dauer TGF-β pathways, which converge on the DA biosynthetic pathway, ultimately regulating the NHR Ce-DAF-12 [38, 41]. Analysis of dauer pathways in the L3i stage of *S. stercoralis* revealed that out of four pathways involved in dauer formation, two were conserved while two were not, suggesting their conserved and novel modes of developmental regulation [41, 67]. Our results show that at least two of the canonical dauer pathways-insulin signaling pathway and cGMP-PKG signaling pathway were represented in the top 60 active pathways by at least 46 and 32 proteins, respectively (Table 3). Further, TGF-β pathway was represented by 27 proteins, and the dafachronic acid pathway was represented by a single but important gene, *daf-1* (Additional file 11: Table S8). DAF-1 encodes a TGF-beta type I receptor homolog, which, in association with the DAF-4, regulates dauer formation in response to environmental signals through the ASI chemosensory neuron [68–70]. Our results show that similar to *C. elegans*, all the four dauer formation pathways are conserved and active in the IJ stage of *H. indica*.

EPN IJs are not known to feed, but they utilize the lipids and glycogen energy reserves stored in the body for their survival. We found genes involved in various pathways like fatty acid degradation, glycolysis, and glyoxalate in the IJ transcriptome. All these three pathways catabolize energy reserves such as fatty acids and glucose and generate ATPs that are utilized for the IJ survival. Glyoxalate pathway has been known to be important for dauer stages of *C. elegans* [71] and has also been reported in an EPN, *Romanomermis* [72].

We found several signaling pathways in the transcriptome of *H. indica* IJs essential for nematode survival under stressed conditions and various other activities (Table 3). Some of these signaling pathways, such as PI3K-Akt and mTOR signaling pathways are involved in regulation of cell cycle and in mediating oxidative stress responses and extending the lifespan in the nematodes [73, 74]. Presence of other signaling pathways such as the MAPK known to be involved in nematode response to various cellular and environmental stimuli including stresses and cell proliferation, regulation of fertilization in nematodes, especially sperm activation [75, 76] suggest that these signaling pathways might control the IJ nematodes from being reproductive in the arrested stage. cGMP-PKG signaling is involved in olfactory sensing and behavior regulation in the nematodes [77, 78] and flies [78, 79], and pharyngeal pumping rate, mouth form dimorphism, the duration of forward locomotion, and the amount of fat stored in the intestine in necromenic insect associated nematode, *Pristionchus* [80]. This indicated that the *H. indica* IJs also actively sense their environment and adapt their metabolism and behavior accordingly.

The analysis of the *H. indica* secretome identified several hydrolases, a large contingent of peptidases, kinases, phosphatases, and enzymes involved in stress responses. Some of these enzymes are important for the degradation of insect cuticle, tissue, and hemocoel, whereas peptidases are also known to be involved in regulatory functions. The presence of a large number of kinases and phosphatases indicates vibrant signaling in the IJ stage. All these findings suggest that although IJ is a developmentally arrested stage; it is still a hotbed of signaling and is actively sensing its environment.

*H. indica* is a rhabditid as *C. elegans*, which shows the presence of 77 RNAi pathway genes [54]. Primary sequence similarity based search was carried out to identify putative orthologs of *C. elegans* RNAi pathway genes in *H. indica.* We found 24 orthologs of *C. elegans* RNAi pathway effector genes in *H. indica* IJs*.* The completed genome sequence of another species of the same genus, *H. bacteriophora* revealed the presence of only 12 RNAi pathway genes [30] indicating either incompleteness of the genome or false negatives because of poor annotation of *H. bacteriophora* genome. Interestingly, the RNAi pathways can differ significantly even amongst very closely related nematode species, as is evident by the fact that the number of RNAi effector genes varied from 60 to 77 amongst different species of *Caenorhabditis spp.* [54]*.* Out of the four RNAi effector genes present in most known parasitic nematodes, *drsh-1*, *rsd-3*, *ego-1,* and *smg-2* were present in *H. indica* IJs. However, *ego-1* was absent in the two parasitic nematodes *Trichinella spiralis*, and *A. caninum* [54], suggesting that it is not universally present in parasitic nematodes as thought earlier. We found *nrde-3* in *H. indica* IJs at a low stringency cutoff, which is responsible for nuclear translocation of RNAi triggers in *C. elegans*, and is involved in processes that lead to the heritability of gene silencing events. The absence of *nrde-3* in parasitic nematodes has led to speculations that silencing events cannot be passed between generations of parasitic nematodes [54]. However, sequences with loose homology to the *C. elegans nrde-3* could be discovered in *H. bacteriophora* genome as well, suggesting that the absence of *nrde-3* in *H. bacteriophora* might be a false negative caused by a failure to predict the *H. bacteriophora nrde-3* gene. Its presence in *Heterorhabditis* nematodes indicated that the silencing events could probably be passed between generations, and opens up a whole new array for use of Heterorhabditid nematodes as a model for epigenetic regulation of RNAi pathways.

The sequence divergence between *C. elegans* and *H. indica* prevented discovery of *C. elegans* orthologs of important gene class members at a high stringency. By lowering the stringency of the blastn to 30 % identity and query coverage, we could identify several additional members of the various gene classes in *H. indica*, but these orthologs would need further validation. The *H. indica* transcriptome showed the presence of at least 22 *flp*, 25 *nlp* and 18 *ins* neuropeptide genes, 69 *unc*, 21 *daf* and 0 (4 at 25 %) *far* genes, 98 *nhr*, nine *lec*, 15 *let* but no *lys* gene class members (Table 7, Additional file 11: Table S8). In the *daf* gene class, *daf-1*, *daf-2* and *daf-4* were identified, all of which are important in dauer formation in *C. elegans. daf-1* encodes a TGF-beta type I receptor homolog, which together with the TGF-β-like type II receptor DAF-4, is required for the regulation of dauer formation by environmental signals [81–84]. Similarly, *daf-7* encodes a member of the TGF-β superfamily; which is involved in signaling pathway that interprets environmental conditions to regulate energy balance pathways that affect dauer larval formation, fat metabolism, egg laying, feeding behavior and sperm motility [85–88]. Identification of several insulin-like peptide (*ins*) genes proved the role of insulin signaling in IJ formation and maintenance in *H. indica*. Neuropeptides like *flp* and *nlp* are involved in environmental sensing by the nematode. In the *flp* gene class, *flp-1*, *flp-3, flp-5, flp-12, flp-17* and *flp-18* were the prominent members. In the recent years, *flp* genes are emerging as important targets for nematode management, and it has been shown that disruption of *flp* gene expression impaired nematode parasite’s ability to locate its host [89–95]. Other neuropeptides found in *H. indica*, like *nlp-4,* has no known homologs in other nematode species [90, 96, 97], whereas *nlp-18* in *C. elegans* encodes four predicted neuropeptide-like proteins; and is expressed in a variety of neurons, spermatheca, the rectal gland, and the intestine [98]. Another important protein class, nematode lectins, are protein molecules that bind to carbohydrate moieties. They are involved in cell-cell recognition and are important in nematode recognition of bacteria and innate immune responses against pathogens. Nine members of the *lec* gene class were identified in *H. indica* including *lec-6. lec-6* encodes a 'proto' type galectin (beta-galactosyl-binding lectin) containing a single carbohydrate recognition domain and is suggested to be important for cell adhesion and aggregation, proliferation, or programmed cell death in *C. elegans* [99–101]. Likewise, in *H. indica*, members of the lectin protein family might possibly be involved in recognition of the symbiont bacteria. Similarly, *tol-1* found expressing in *H. indica* IJs has been reported to be involved in behavioral responses to the pathogenic microbes by promoting the development of sensory neurons that monitor microbial metabolism and are required for a pathogen-avoidance behavior in *C. elegans* [102]. Hence, it is possible that *tol-1* could be involved in the maintenance of a specific symbiotic relationship between *Heterorhabditis* nematodes with *Photorhabdus* bacterium, but this hypothesis would need further testing.

## Conclusions

Here we presented a transcriptomic insight into the infective juvenile stage of the EPN, *H. indica*. After using cd-hit-est and filtering out <300 bp transcripts, we have identified 13,592 unique transcripts in *H. indica* infective juveniles. 18.6 % of the proteins were similar to an animal parasite *A. suum*. We found that similar to *C. elegans*, all the four dauer formation pathways-cGMP-PKG signaling pathway, insulin signaling pathway, dafachronic acid pathway, and TGF-β were conserved in *H. indica* and were active in the IJ stage of the nematode. Several important signaling pathways were found active in the IJs indicating that despite being a developmentally arrested stage, IJs are a hotbed of signaling and are actively interacting with their environment. Similarly, glycolysis and fatty acid degradation pathways were highly active in IJs indicating a breakdown of food reserves required for survival. Twenty-four orthologs of *C. elegans* RNAi pathway effector genes were found in *H. indica* IJ transcriptome, including *nrde-3* that has been identified in any of the parasitic worms for the first time. Using a low stringency approach, we have identified several additional members of important gene classes in *H. indica*. Our results and analysis lay down the groundwork for further functional genomic investigations on these gene classes in *Heterorhabditis* nematodes.

## Methods

### Nematode collection and multiplication

The *Heterorhabditis indica* nematodes were isolated from the soil collected from Ghaziabad district, UP, India by using greater wax moth *Galleria melonella* as a bait. The nematodes were maintained in the laboratory on *Galleria* using standard procedures.

### RNA extraction, cDNA synthesis, library preparation and sequencing

Total RNA was extracted from the frozen IJs using Nucleospin RNA isolation kit (Macherey-Nagel GmbH & Co. KG, Düren, Germany) according to the manufacturer’s instructions. Extracted RNA was assessed for quality and quantity using an Agilent 2100 Bioanalyzer (Agilent Technologies). RNA with an RNA integrity number (RIN) of 8.0 was used for mRNA purification. mRNA was purified from 1 mg of intact total RNA using oligodT beads (Illumina® TruSeq® RNA Sample Preparation Kit v2). The purified mRNA was fragmented at elevated temperature (90 °C) in the presence of divalent cations and reverse transcribed with Superscript II Reverse Transcriptase (Invitrogen Life Technologies) by priming with random hexamers. Second strand cDNA was synthesized in the presence of DNA polymerase I and RNaseH. The cDNA was cleaned using AgencourtAmpure XP SPRI beads (Beckman-Coulter). Illumina adapters were ligated to the cDNA molecules after end repair and the addition of an ‘A’ base followed by SPRI clean-up. The resultant cDNA library was amplified using PCR for the enrichment of adapter-ligated fragments, quantified using a Nanodrop spectrophotometer (Thermo Scientific) and validated for quality with a Bioanalyzer (Agilent Technologies). It was then sequenced on the Illumina Hiseq 2000 platform at SciGenom Next-Gen sequencing facility, Cochin, India. Both the raw and assembled sequence data generated has been deposited in the European Nucleotide Archive (ENA) database (http://www.ebi.ac.uk/ena) for public access (raw data accession no.: PRJEB10852, assembled contigs accession numbers: HADG01000001-HADG01013593). The assembled nucleotide and protein sequences are also available for blast and download at http://insilico.iari.res.in/hindica/. The assembled data is included with the manuscript as Additional file 12.

### De novo transcriptome assembly and analysis

Paired orphan sequence reads obtained from IJs were used for assembly of the transcriptome [103]. The low quality reads (Phred score <30) were removed and sequencing statistics was generated with the help of NGSQC Toolkit version v2.3.3 [104]. High quality filtered paired-end raw reads (Phred Score ≥ 30) obtained from IJs were assembled using Velvet (v.1.2.08) and Oases (v.0.2.08) pipeline [105]. Velvet was run at different k-mer lengths (51–93 with a step size of 4)—with minimum contig length of 200. The optimal assembly was attained at k-mer 83. The oases module was used for merging transcript assemblies from k-mer 71 to 83 (71, 75, 79, 83) with minimum transcript length of 100 using the script “oases_pipeline.py” (k-mer range 71–83, insert length 250 bp, coverage depth cut off 5). Cd-hit-est was used to remove redundant transcripts at 90 % similarity. Transcripts <300 nucleotide length were removed resulting in a unique set of non-redundant transcripts.

### Annotation and quantification of the transcriptome

ORFPredictor web server (http://bioinformatics.ysu.edu/tools/OrfPredictor.html) [51] was used to predict proteins from the 13,593 transcripts (>300 bp length) using the default cut-off value of 1e–5, and 13,592 proteins were predicted which were used for annotation. Annotation for all the unique transcripts (>300 bp) was done using blastp [106], homology search against Uniprot [107], the National Center for Biotechnology Information (NCBI)-NR Protein database [106] and NEMABASE4 (http://www.nematodes.org/nembase4/). In addition, blastx was performed to identify homologues at ≥30 % query coverage and ≥50 % sequence identity and e-value 1e–5 in other databases including RefSeq (PRK), SWISSPROT [108], European Molecular Biology Laboratory(EMBL)[109], DNA Databank of Japan (DDBJ) [110], Protein Information Resource (PIR) [111] and Protein Data Bank (RCSB)[112]. Nematode orthologs were identified from NCBI COG [113] database and other completely sequenced genomes by the reciprocal blast method[106]. To study gene orthologs across free-living and parasitic nematode species, we used the predicted protein sets from 11 genomes available in the public domain (Wormbase, NCBI, and Sanger) viz., *C. elegans, C. remanei, C. briggsae, M. hapla, M. incognita, H. bacteriophora, Pristionchus pacificus, Brugia malayi, S. ratti, Trichinella spiralis* and *A. suum*. Blastp hits with e-value scores 1e–5 and query coverage above 50 % were considered as annotated homologous proteins and python script was employed for filtering reciprocal best hits. KEGG orthologs were identified using the KEGG Automated Annotation Server (KAAS) using nematode database. iPATH server was used for mapping it to KEGG reference pathway [114]. The gene ontology and domains were identified using InterProScan 5 with default parameters [115]. The resulting hits were processed to retrieve associated GO terms describing biological processes, molecular functions, and cellular components. Homologs of the *C. elegans* RNAi pathway genes were also identified in the *H. indica* transcriptome by performing tblastx with e-value ≤ 1e–5.

The high-quality reads were mapped to the non-redundant assembled transcripts using TopHat v-2.0.9. [116–119]. Assembly of transcript models from RNA-Seq alignments and estimation of transcripts and their abundance was performed using Cufflinks v-2.1.1 [119]. Both these software packages were used with default parameters for our analysis [119].

Potentially secreted peptides were identified using the SignalP 4.1 software [120] from the 174,700 peptides of minimum protein length ≥30, and those with transmembrane motifs were removed using TMHMM [121]. MEROPS database was searched to identify proteases, proteinases, and proteolytic enzymes [122]. Repeat elements were identified in transcripts using Repeat Masker v.4.0.5 S and Repbase v.20140131 using default parameters against species “Nematoda”. Short Sequence Repeats (SSRs) were identified using MISA (MIcroSAtellite; http://pgrc.ipk-gatersleben.de/misa) with at least 10 repeats for mono-, 6 repeats for di-, and 5 repeats for tri-, tetra-, penta- and hexanucleotide for simple SSRs.

## Availability of supporting data

The data supporting the results of this research paper are included within this article and its additional files. The raw and assembled sequence data has been deposited in the Eueopean Nucleotide Archive (ENA) database for public access (raw data accession no.: PRJEB10852, assembled contigs accession numbers: HADG01000001-HADG01013593). The assembled nucleotide and protein sequences are available for blast and download at http://insilico.iari.res.in/hindica/. The assembled sequences are also supplied as an Additional file 12 with this manuscript.

## Additional files

Additional file 1: Table S1a. Blast, KOG and PRK analysis of the *H. indica* IJ transcripts. (XLSX 2073 kb) Additional file 2: Table S1b. Standalone blast of *H. indica* IJ transcripts against *H. bacteriophora* genome. (XLSX 336 kb) Additional file 3: Table S2. Transcript abundance of *H. Indica* transcripts along with KOG analysis of transcripts showing FPKM values ≥ 100 and ≥ 1000. (XLSX 486 kb) Additional file 4: Table S3. KEGG Pathways represented by transcripts with FPKM value of ≥100 and ≥1000. (XLSX 20 kb) Additional file 5: Table S4.
*C. elegans* kinase Group/family/subfamily not found in *H. indica.* (XLSX 10 kb) Additional file 6: Table S5a.
*H. indica* secretome prediction by SignalP. (XLSX 90 kb) Additional file 7: Table S5b. MEROPS analysis of *H. indica* transcripts to identify secreted peptidases, proteases, and peptides. (XLSX 11 kb) Additional file 8: Table S6a. Repeat elements identified in *Heterorhabditis indica* transcripts. (DOCX 16 kb) Additional file 9: Table S6b. SSR type and microsatellites present in *H. indica.* (XLSX 187 kb) Additional file 10: Table S7. RNAi effector pathway genes in *H. indica.* (XLSX 12 kb) Additional file 11: Table S8. Orthologs of members of *C. elegans* gene classes present in *H. indica* at ≥25% identity and query coverage. (XLSX 37 kb) Additional file 12:
*H. indica* assembled sequences. (TXT 14804 kb)
