# Supplementary material for: A transcriptomic insight into the infective juvenile stage of the insect parasitic nematode, Heterorhabditis indica
Source: BMC Genomics. 2016 Mar 1;17:166. doi: 10.1186/s12864-016-2510-z (PMC4774024; doi:10.1186/s12864-016-2510-z)
Supplement: Additional file 8: Table S6a. — Repeat elements identified in Heterorhabditis indica transcripts. (DOCX 16 kb) [file 12864_2016_2510_MOESM8_ESM.docx]

| **Repeat Type** | **Number of element** | **Length occupied** | **Percentage of sequence** |
| --- | --- | --- | --- |
| **Retro elements** | 31 | 1102 bp | 0.01 % |
| SINEs | 0 | 0 bp | 0.00 % |
| Penelope | 1 | 107 bp | 0.00 % |
| LINEs | 4 | 271 bp | 0.00 % |
| CRE/SLACS | 0 | 0 bp | 0.00 % |
| L2/CR1/Rex | 2 | 128 bp | 0.00 % |
| R1/LOA/Jockey | 1 | 36 bp | 0.00 % |
| R2/R4/NeSL | 0 | 0 bp | 0.00 % |
| RTE/Bov-B | 0 | 0 bp | 0.00 % |
| L1/CIN4 | 0 | 0 bp | 0.00 % |
| LTR elements: | 27 | 831 bp | 0.00 % |
| BEL/Pao | 24 | 686 bp | 0.00 % |
| Ty1/Copia | 0 | 0 bp | 0.00 % |
| Gypsy/DIRS1 | 3 | 145 bp | 0.00 % |
| Retroviral | 0 | 0 bp | 0.00 % |
| **DNA transposons** | 15 | 1517 bp | 0.01 % |
| hobo-Activator | 0 | 0 bp | 0.00 % |
| Tc1-IS630-Pogo | 8 | 1097 bp | 0.01 % |
| En-Spm | 0 | 0 bp | 0.00 % |
| MuDR-IS905 | 0 | 0 bp | 0.00 % |
| PiggyBac | 0 | 0 bp | 0.00 % |
| Tourist/Harbinger | 0 | 0 bp | 0.00 % |
| Other (Mirage, P-element, Transib) | 0 | 0 bp | 0.00 % |
| **Rolling-circles** | 0 | 0 bp | 0.00 % |
| **Unclassified:** | 0 | 0 bp | 0.00 % |
| **Total interspersed repeats:** |  | 2619 bp | 0.01 % |
| **Small RNA:** | 103 | 38139 bp | 0.18 % |
| **Satellites:** | 3 | 104 bp | 0.00 % |
| **Simple repeats:** | 5709 | 251409 bp | 1.21 % |
| **Low complexity:** | 1036 | 60116 bp | 0.29 % |

**Table S6a.** Repeat elements identified in *Heterorhabditis indica* transcripts

*most repeats fragmented by insertions or deletions have been counted as one element. The query species was assumed to be Nematoda. Repeat Masker version open-4.0.5, default mode run with rmblastn version 2.2.23+ RepBase Update 20140131, RM database version 20140131
